# Supplementary material for: Extracellular Overexpression of a Neutral Pullulanase in Bacillus subtilis through Multiple Copy Genome Integration and Atypical Secretion Pathway Enhancement
Source: Bioengineering (Basel). 2024 Jun 28;11(7):661. doi: 10.3390/bioengineering11070661 (PMC11273987; doi:10.3390/bioengineering11070661)
Supplement: Supplementary file 1 [file bioengineering-11-00661-s001.zip › bioengineering-3073670-supplementary.pdf]

bioengineering

MDPI

Supplementary Materials

Extracellular Overexpression of a Neutral Pullulanase in *Bacillus subtilis* through Multiple Copy Genome Integration and Atypical Secretion Pathway Enhancement

Wenkang Dong [1,†], Xiaoping Fu [2,3,4,†], Dasen Zhou [1], Jia Teng [3], Jun Yang [3], Jie Zhen [2,3,4], Xingya Zhao [2,3,4], Yihan Liu [1], Hongchen Zheng [2,3,4,5,*] and Wenqin Bai [2,3,4,5,*]

Table S1. Plasmids used in this study.

Plasmids	Properties	reference
pMA05-*pulA3E*	Expression plasmid, AmpR (*E. coli*), KanR (*B. subtilis*), *pulA3E* (encoding gene of PulA mutant R503E/I506E/H507E)	In this work
pMA05-0046*ytnA*	Expression plasmid, Amp*r* (*E. coli*), KanR (*B. subtilis*), transporter encoding gene 0046*ytnA*	In this work
pMA05-*pulA3E*-0572*comEA*	Expression plasmid, AmpR (*E. coli*), KanR(*B. subtilis*), transporter encoding gene 0572*comEA*	In this work
pMA05-pulA3E-2143spoIIQ	Expression plasmid, AmpR (*E. coli*), KanR (*B. subtilis*), transporter encoding gene *spoIIQ*	In this work
pMA05-*pulA3E*-3038*yvbW*	Expression plasmid, AmpR (*E. coli*), KanR (*B. subtilis*), transporter encoding gene 3038*yvbW*	In this work
pMA05-*pulA3E*-0572*comEA*-RBS-3038*yvbW*	Expression plasmid, AmpR (*E. coli*), KanR (*B. subtilis*), encoding gene of 0572*comEA*-*RBS*-3038yvbW expression box	In this work
pHT43-cas9	Rigorous expression plasmid, AmpR (*E. coli*), CamR (*B. subtilis*), *cas9* gene	In this work
pUC980-2-*amyE*-N20-up-down-*pulA3E*	High-copy shuttle plasmid, AmpR (*E. coli*), KanR (*B. subtilis*), amyE-N20, upstream and downstream homologous sequences of *amyE*, *pulA3E*	In this work
pUC980-2-*ytxE*-N20-up-down-*pulA3E*	High-copy shuttle plasmid, AmpR (*E. coli*), KanR (*B. subtilis*), *ytxE*-N20, upstream and downstream homologous sequences of *ytxE*, *pulA3E*	In this work
pUC980-2-*ytrF*-N20-up-down-*pulA3E*	High-copy shuttle plasmid, AmpR (*E. coli*), KanR (*B. subtilis*), *ytrF*-N20, upstream and downstream homologous sequences of *ytrF*, *pulA3E*	In this work
pUC980-2-*trpP*-N20-up-down-*pulA3E*	High-copy shuttle plasmid, AmpR (*E. coli*), KanR (*B. subtilis*), *trpP*-N20, upstream and downstream homologous sequences of *trpP*, *pulA3E*	In this work
pUC980-2-*nprB*-N20-up-down-*pulA3E*	High-copy shuttle plasmid, AmpR (*E. coli*), KanR (*B. subtilis*), *nprB*-N20, upstream and downstream homologous sequences of *nprB*, *pulA3E*	In this work

Table S2. Primers used in this study.

Primer name	Sequence (5'-3')
MA05-F	ATGTAAATCGCTCCTTTTTAGG
MA05-R	GCTAGCTTGGTACGTACC
amyE-N20-F	CAGTGATAGCCTGATCTTCAACATTTATTGTACAACACGAGC

amyE-N20-R	TGAAGATCAGGCTATCACTGGTTTTAGAGCTAGAAATAGCAA
ytxE-N20-F	ATACCGATACCCGTCGAACTACATTTATTGTACAACACGAGC
ytxE-N20-R	AGTTCGACGGGTATCGGTATGTTTTAGAGCTAGAAATAGCAA
ytrF-N20-F	ATACCGATACCCGTCGAACTACATTTATTGTACAACACGAGC
ytxE-N20-R	AGTTCGACGGGTATCGGTATGTTTTAGAGCTAGAAATAGCAA
ytrF-N20-F	TTGGTTGTGTTGTCGTCCGTACATTTATTGTACAACACGAGC
ytrF-N20-R	ACGGACGACAACACAACCAAGTTTTAGAGCTAGAAATAGCAA
trpP-N20-F	ATTATTCCGCCCTTTTTAGGACATTTATTGTACAACACGAGC
trpP-N20-R	CCTAAAAAGGGCGGAATAATGTTTTAGAGCTAGAAATAGCAA
nprB-N20-F	GGCCAGTAATAGAGATGTCTACATTTATTGTACAACACGAGC
nprB-N20-R	AGACATCTCTATTACTGGCCGTTTTAGAGCTAGAAATAGCAA
MA05-HF	GTTGTTTTGGGGGCATCATAtgtaaatcgctccttttaggtgg
MA05-HR	TTAATTCAATGTTGAggatcctctagagtcgagctc
pulA3E-F	ATGCCCCCAAAACAACAG
pulA3E-R	TCAACATTGAATTAATACCCACG
MA05-PF	TTTTTGAGAAGATCACTCAAAAAATC
MA05-PR	ATGCCCCCAAAACAACAGT
P_{cry3A}-F	CTTCTCAAAAAATACGGCCTTTGAATTGTAACGCC
P_{cry3A}-R	TTGTTTTGGGGGCATTCGGTTCCCTCCTCACTTTTC
amyE-up-F	TTCTGTACAAATCTTTAACAAAATTCTCCAGTCTTCAC
amyE-up-R	TATCGGCCGAAGCTTCTAGGATCCGATCAGACCA
amyE-down-F	TTAATTCAATGTTGATCGACATGGATGAGCG
amyE-down-R	ACGCGTCCATGGAGATTCAATGGGGAAGAGAACCG
ytxE-up-F	CTTCTGTACAAATCTCTGACCTCTTTCTTCATCGTAAC
ytxE-up-R	CGTTACAATTCAAAGTGCAGATCGATTTGGG
ytxE-down-F	CCAGTTTGGCGCGCCGGATAACAAGACAAATGAACAC
ytxE-down-R	CGCGTCCATGGAGATGAGAGCATCAGGATTCTGC
ytrF-up-F	CTTCTGTACAAATCTGCCGGTTATCTCATTGG
ytrF-up-R	TTACAATTCAAAGGCCACCTGATCCTTAAACCTCAAC
ytrF-down-F	CCAGTTTGGCGCGCCCGGCGAGAAAAGCTACC
ytrF-down-R	CGCGTCCATGGAGATCCGCCTTTCGATAAGGAATG
trpP-up-F	CTTCTGTACAAATCTATTCCAGAGGTTGACCTT
trpP-up-R	TACAATTCAAAGGCCTGCGGCAAACAGAGCCA
trpP-down-F	TCCAGTTTGGCGCGCCCCGATTGTGCAAACCATTTTAA
trpP-down-R	CGCGTCCATGGAGATGCGACGAAAGGGTTTTTC
nprB-up-F	CTTCTGTACAAATCTCGAAGATGCAGTCACAATTCATGC
nprB-up-R	TTACAATTCAAAGGCCACGGCATCTACAGATTTCAGCATGATG
nprB-down-F	TCCAGTTTGGCGCGCCGCGCAAAACACCACATCCTTCC
nprB-down-R	CGCGTCCATGGAGATGGAACTGCAGGAAGCTG
yvbw-F	ATCGCTCCTTTTTAGGATGAAAAACGACAATCAAACG
yvbw-R	TAGCTTGGTACGTACCTTACTGATGCTTGCGTCCTTTG
ytnA-F	cctaaaaaggagcgatttacaTATGATGCAAAAACAAAAACAAGAGCTG
ytnA-R	gagctcgactctagaggatccTCAGCTGATATTTCGTTCGCTG
comEA-F	ATCGCTCCTTTTTAGGATGAATTGGTTGAATCAGCATAAG
comEA-R	TAGCTTGGTACGTACCTCACTTTACTGTAATGGAAGAC
spoIIQ-F	ATCGCTCCTTTTTAGGATGAGAGAGGAAGAAAAGAAAAC
spoIIQ-R	TAGCTTGGTACGTACCTTAAGACTGTTCAGTGTCTTCTG
yvbw-F1	cctaaaaaggagcgatttacaTATGATGAAAAACGACAATCAAACGTTAAAA
yvbw-R1	gagctcgactctagaggatccTTACTGATGCTTGCGTCCTTTGA
MA05-CF	ggatcctctagagtcgagctc
MA05-CR	CATAtgtaaatcgctccttttaggtgg

MA05-*comEA*-F	CATTACAGTAAAGTGAaaaggagcgatttacaTATGATGAAAAA
MA05-*comEA*-R	ggatcctctagagtcgagctcaag
yvbW-PF	CATTACAGTAAAGTGAaaaggagcgatttacaTATGATGAAAAA
yvbW-PR	gagctcgactctagaggatccTTACTGATGCTTGCGTCCTTTGA

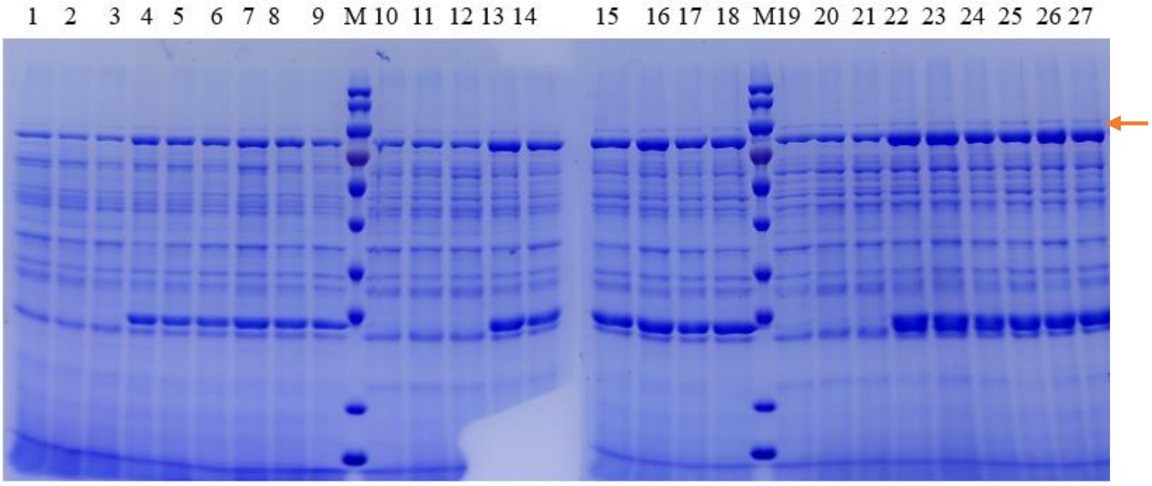

Figure S1. SDS-PAGE of the extracellular proteins of the genome integration engineered strains at different fermentation time. Band 1,2,3: the extracellular proteins of PA after 24 h fermentation; Band 4,5,6: the extracellular proteins of PB after 24 h fermentation; Band 7,8,9: the extracellular proteins of PB-2 after 24 h fermentation; Band 10,11,12: the extracellular proteins of PA after 48 h fermentation; Band 13,14,15: the extracellular proteins of PB after 48 h fermentation; Band 16,17,18: the extracellular proteins of PB-2 after 48 h fermentation; Band 19,20,21: the extracellular proteins of PA after 96 h fermentation; Band 22,23,24: the extracellular proteins of PB after 96 h fermentation; Band 25,26,27: the extracellular proteins of PB-2 after 96 h fermentation; Band M: the protein molecular weight markers, the bands from top to bottom represent 180kDa, 130kDa, 95kDa, 70kDa, 55kDa, 43kDa, 33kDa, 25kDa, 17kDa, and 10kDa, respectively.

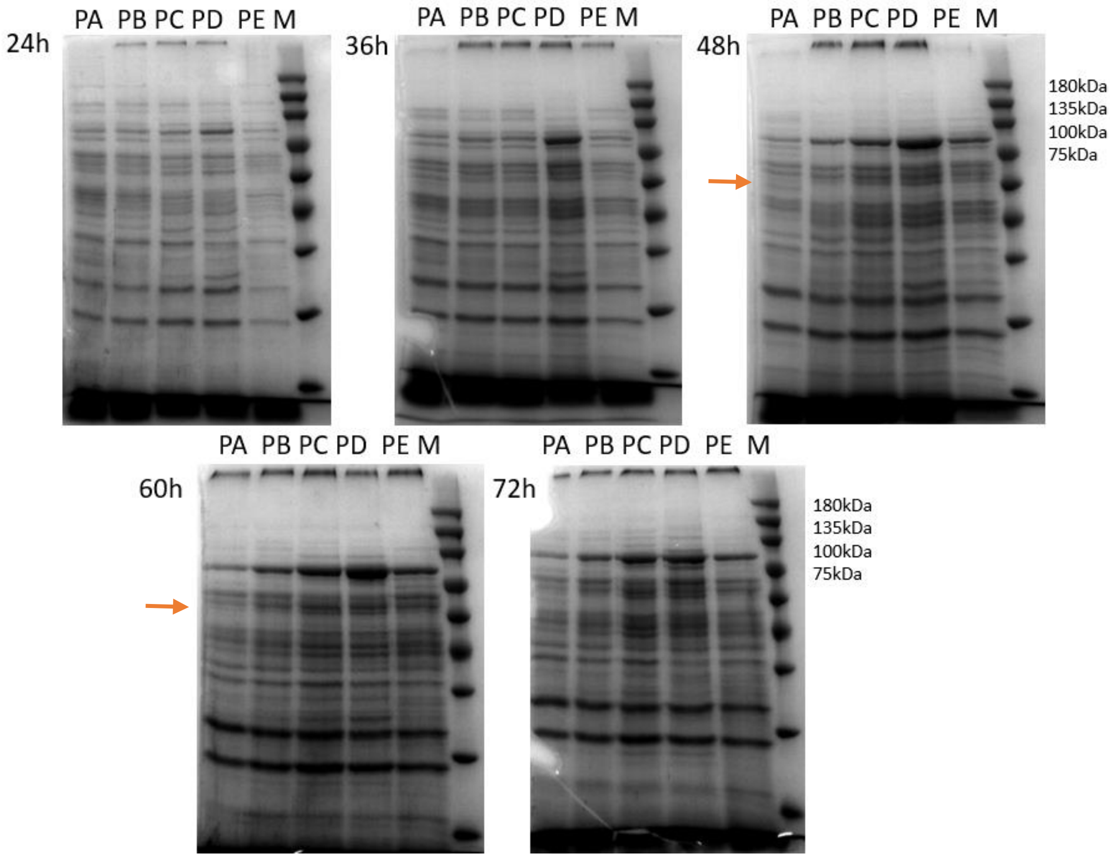

Figure S2. SDS-PAGE of the extracellular proteins of the genome integration engineered strains with 1-4 copies of PulA3E at different fermentation time.

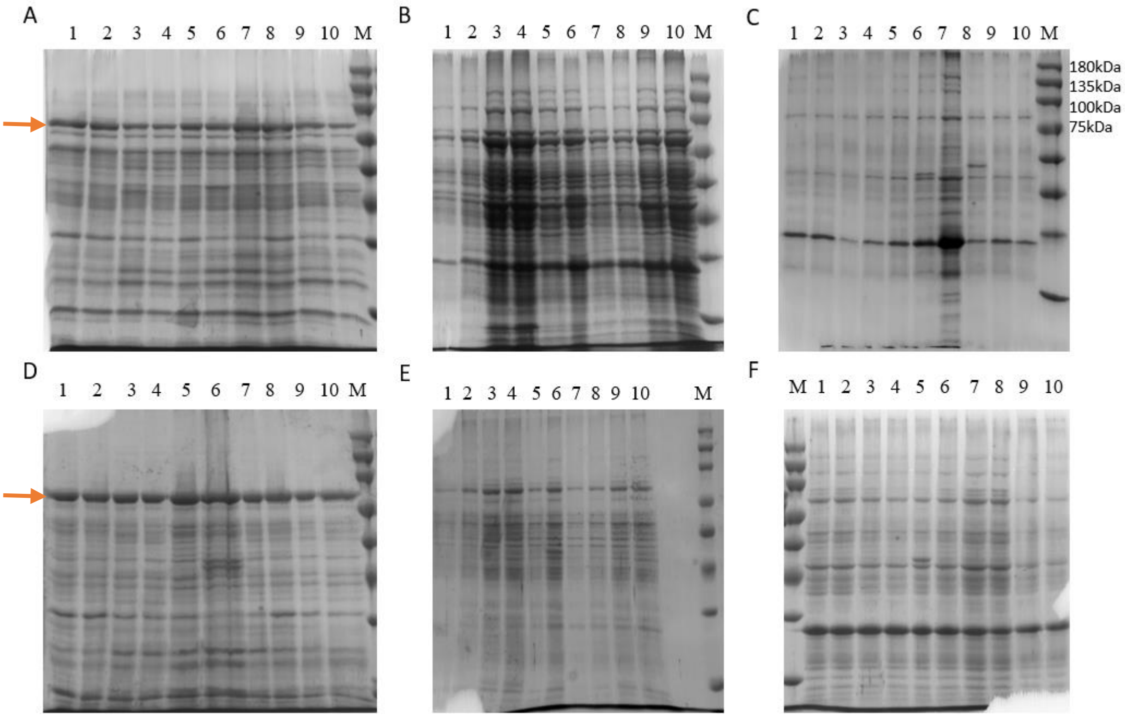

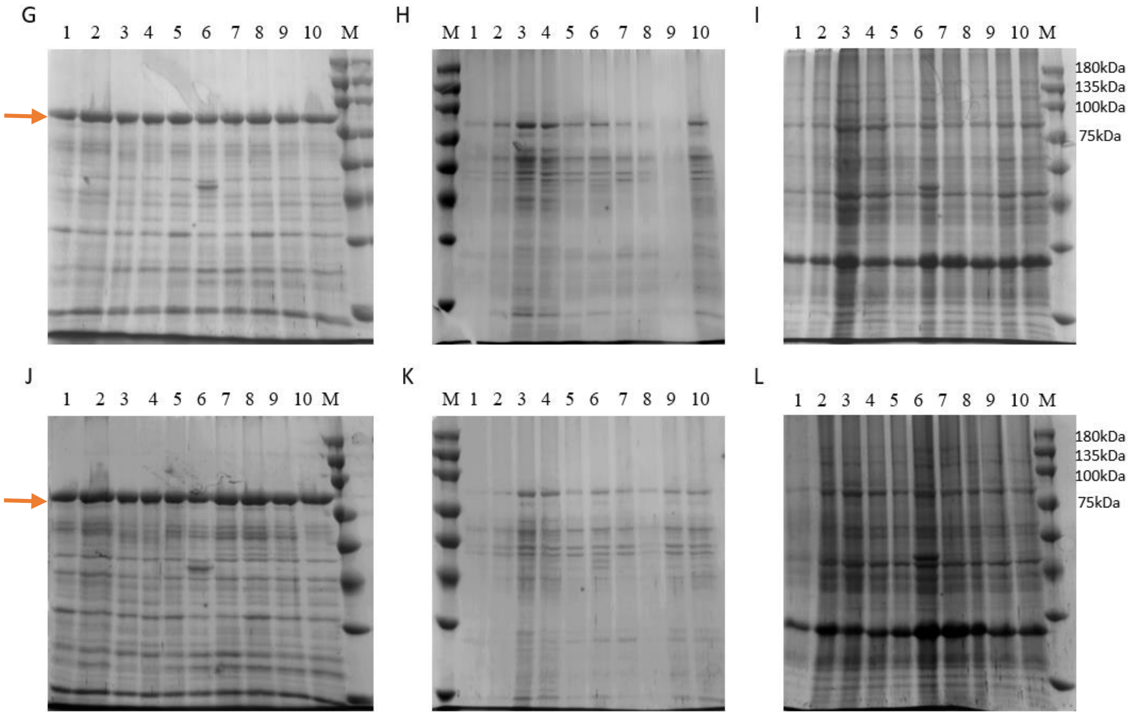

Figure S3. SDS-PAGE of the proteins of the engineered strains with the overexpression of different transporters for the PulA-type atypical secretion pathway at different fermentation time. A,D,G,J: the extracellular proteins; B,E,H,K: the intracellular proteins; C,F,I,L: the inclusion body; A,B,C: 24h fermentation; D,E,F: 48h fermentation; G,H,I: 72h fermentation; J,K,L: 96h fermentation; 1,2: overexpression of *ytnA* in PD; 3,4: overexpression of *comEA* in PD; 5,6: overexpression of *spoIIQ* in PD; 7,8: overexpression of *yvbW* in PD; 9,10: the control strain PD; M: the protein molecular weight markers.
